# Birmingham Hospital Saturday Fund

**Published:** 1894-05-12

**Authors:** 


### THE INSTITUTIONAL WORKSHOP.

### HOSPITAL ADMINISTRATION.

### BIRMINGHAM HOSPITAL SATURDAY FUND.

This fund has attracted a great deal of notice in consequence of the vigour which has characterised its management for some years past, the relative largeness of the sum which has been annually collected, and the claim which the Committee put forth that all the money raised is given as a free gift to the Birmingham hospitals. The Committee have further determined that £10,000 is an adequate annual sum for the working classes to contribute directly to its hospitals on Hospital Saturday. They have also initiated a policy by which they have established a Convalescent Home for working-men at Tyn-y-Coed, near Llandudno, which includes a model farm and a market garden, the whole of which were presented to the Fund by the Misses Stokes, in memory of their late brother. In addition to this, the Committee have taken on lease Marle Hall, with its furniture, and about 34 acres of land, which was opened as a Convalescent Home for Women by the Mayor of Birmingham, on Saturday, May 5th.

Notable features of these homes are their non-institutional character, the beauty of their surroundings, the system upon which they are conducted, and the educational means which they undoubtedly supply for the elevation and the moral improvement of all who are fortunate enough to become inmates of them. A careful inspection of these homes has convinced us that there is nothing like them in this country. The beauty of the gardens and grounds, and their extent and character, the fact that the furniture and decorations are chaste and artistic, and that the inmates enjoy home comforts to an extent not heretofore present in convalescent institutions, must all tend to elevate those who enter them, and make them acquainted with simple joys hitherto unrealised and unknown to most dwellers in the poorer parts of our large cities. All honour, therefore, to the working men of Birmingham, and to the Committee of the Hospital Saturday Fund, for the wisdom and courage they have displayed in establishing these homes, and the excellence of all the arrangements connected with them.

Mr. Burdett was specially invited to the opening of Marle Hall, on Saturday, and was entrusted with the toast of "Success to the Birmingham Hospital Saturday Fund." After giving an account of the early history of the Fund, and expressing the opinion that the Birmingham Hospital Saturday Fund was the most successful and the most fruitful in interest of all Hospital Saturdays, Mr. Burdett proceeded to impress upon those present the great responsibility which so successful a movement imposed upon the Executive Committee. He pointed out that the working men of Wolverhampton contributed to the hospitals every year £39 per thousand of the population; West Bromwich, £35 per thousand; Coventry, £25 per thousand; and Birmingham, £21 10s. per thousand. He then went on to state that with the exception of the Birmingham Dental Hospital, where attendances only are given, and the Birmingham Hospital for Women, of which no particulars were forthcoming, the remaining ten hospitals and dispensaries in Birmingham relieved 163,994 out-patients, at a cost of £16,870, and 8,944 in-patients, at a cost of £28,862, in the year 1892. In other words, the total expenditure upon patients by the hospitals in question amounted to £45,732 in 1892, in which year £11,908 was raised and £11,000 distributed amongst the hospitals by the Birmingham Hospital Saturday Fund. Hence the principal medical institutions in Birmingham in 1892 expended £45,732 on relief to patients, whereas they received £11,000 from the Hospital Saturday Fund as a free gift, leaving £34,732 to be raised by the hospital committees from other sources. Mr. Burdett expressed a hope that these figures would be carefully examined, and that the Hospital Saturday Fund Committee would ascertain how much of this £34,732 of expenditure was contributed by the working classes in direct subscriptions to the hospitals. He further urged that until the accounts of the Hospital Saturday Fund included the whole of the contributions made by the working classes to the hospitals from every source, it was open to question how far, if at all, the £11,000 could be regarded as a free gift, although no tickets were actually issued in exchange for this money.

It must be admitted that an after-luncheon speech on a fine day, in a tent, is not an occasion which anyone would choose as the one best suited for a discussion upon such questions as those which were raised by Mr. Burdett. At the same time, when an expert is invited to take part in proceedings of this kind, and in reply to a definite inquiry, is requested to speak on the subject of the Hospital Saturday movement, if the speaker has any sense of responsibility, he has no choice if he is to do his duty and be of use to his hosts and their supporters, but to direct attention to material points, and seek for information upon those which appear obscure.

It is perhaps not surprising, though it is certainly regrettable, that Mr. Burdett's inquiries should have been resented somewhat warmly by the Chairman of the Committee, the Hon. Secretary, and some of the Birmingham newspapers. These speakers attributed ignorance to Mr. Burdett, and urged that he was unaware of the large sum that was given direct from the workshops for the purchase of tickets, altogether irrespective of the money given for the free use of the charities. It was maintained that the magnificent sum raised on Hospital Saturday was given after provision had been made for the hospital wants of the employés in the respective workshops. In these circumstances it was urged that a comparison between the amount given in Birmingham and in neighbouring towns per thousand of the inhabitants, was a criticism which was rather harsh than kind. Mr. Smedley the Hon. Secretary, went so far as to say that the £39 per thousand given by Wolverhampton was copper, whereas the £21 10s. given by Birmingham was gold. This was so because the Wolverhampton Hospital Saturday Fund was not a Saturday Fund at all in the sense of the

Birmingham Fund, it was a branch of the infirmary. We imagine that Mr. Smedley must have been unaware that the Wolverhampton Fund is worked by a large special committee, elected by the workshops exactly as in Birmingham, the only difference being, so far as we can discover, that, in addition to fifty-seven working-men members, twelve members of the Weekly Board of the Hospital are *ex-officio* members of it. Mr. Smedley's meaning is therefore a little obscure, and requires elucidation in the interest of the fund which he so ably controls. Perhaps the fact that tickets for the hospitals are issued to the Hospital Saturday Committee in Wolverhampton makes the £39 per thousand, however, in Mr. Smedley's view, nothing better than copper.

Mr. Smedley further dissented from the statement that £11,000 was the contribution of the working men, and that they received £45,732 worth of benefit for it. He declared, indeed, "They did not do anything of the sort, they contributed £25,000 to begin with." We must ask Mr. Smedley to give us some details of these figures, as a careful examination of the reports of the Birmingham hospitals seems to negative them entirely. Taking the accounts for the year 1892 of the Birmingham General, Queen's, Sick Children, Eye, Ear and Throat, Dental, and Skin and Lock hospitals, it appears that the whole sum received by them from every source on account of tickets sold was below £422. Again, the whole of the annual subscriptions received by the same hospitals during the same year, from every source, amounted to only £13,201. Now the reports show that only a proportion of these annual subscriptions (we imagine from a rough estimate that 33 per cent. would be the outside sum), was contributed by the working classes. Let us put it at £4,500, and add to it the whole of the tickets sold, £422, and we then get rather less than £5,000 per annum. This leaves £20,000 of the amount which Mr. Smedley claims to have been paid direct to the hospitals by the working classes yet to be accounted for, and it certainly could not have been given to the General Dispensary, seeing that the whole expenditure of that institution, and the lying-in charity together in 1892 was under £6,000. Will Mr. Smedley kindly supply the facts on which his statement was based?

Once again it appears that in-patients' tickets may be obtained from the General Hospital for a subscription of £2 2s., whereas each in-patient costs £2 10s. 2d. at that institution, and £3 8s. 9d. at the Queen's Hospital; that is an average cost of £3 per in-patient. Hence every in-patient note which is issued by a subscriber entails a loss upon the institution, on the average, of 18s. per patient admitted. This fact is well worthy of the notice of the hospital authorities, as well as those of the Hospital Saturday Fund, and we have no doubt that they will give due attention to the matter, having regard to the statement made on Saturday that the working classes are in the habit of purchasing large numbers of tickets from the hospitals in Birmingham.

No doubt a knowledge of the foregoing facts induced Mr. Burdett to urge the desirability of the adoption by Birmingham of the Potteries' system, by which a record is kept between the subscribing factory and the hospital. This is easily accomplished by issuing to the workshops a number of books containing letters of introduction. These letters are taken to the hospitals by the patients, and the hospital authorities keep a record of the number of cases sent in by each workshop, and of the cost these cases entail. Under such a system there is no rough estimate or bold assertion, but the precise facts are ascertained and known to the hospital authorities and also to the working men too. The result is that in the Potteries the working classes not only pay to the hospitals the actual cost of all the cases they send there for treatment, but they contribute in addition a large sum every year as a free gift towards the maintenance of those members of the community who are less fortunately circumstanced than themselves. What objection can there be to such a business-like arrangement? Certainly not the one advanced by the *Birmingham Daily Mail*, which urges that "each Birmingham factory does not wish to know what return it gets from each hospital from the free gift it makes on Hospital Saturday!" But how can any workshop claim that it makes "a free gift" at all until it has adopted the Potteries' system, and really knows and can prove to demonstration how much cost the hospitals of Birmingham have been put to on account of the cases each workshop has sent to them for treatment during a given year? It is all very well to talk of "a higher ideal, and a loftier practice," but it seems to us it would be a little juster and more in accordance with the common sense and practice of the working classes of Birmingham, that they should put the matter at once upon a business footing, and so ascertain whether they may not be labouring, after all, under a serious mistake in regard to the nature of the magnificent collections which they have undoubtedly made in support of the Birmingham hospitals.

We have been induced to call attention to these questions because we regard the Birmingham Hospital Saturday Fund as a pioneer fund. It has been excellently administered; its committee have shown enterprise, sound judgment, and wise economy in its development, as well as in the management of their Convalescent Homes, and we should look upon it as a calamity if any discredit should attach itself to so excellent a movement for want of precise figures, and the courage to ascertain and look actual facts in the face. By all means let Mr. Burdett be criticised for the physiological outrage he committed in making awkward suggestions and inquiries after a luncheon in a tent, but when he has received sufficient castigation we hope those concerned will turn to the more pressing and important matter. We may then hope as a result that the statements made on Saturday in answer to those inquiries, will either be justified by the production of detailed figures and facts, or that they will be altogether withdrawn, as we make no doubt they will be gladly withdrawn by the speakers, should they find that they were made in error, as we believe them to have been, for the reasons we have already stated.

